# Identification of a public CDR3 motif and a biased utilization of T-cell receptor V beta and J beta chains in HLA-A2/Melan-A-specific T-cell clonotypes of melanoma patients

**DOI:** 10.1186/1479-5876-7-21

**Published:** 2009-03-24

**Authors:** Federico Serana, Alessandra Sottini, Luigi Caimi, Belinda Palermo, Pier Giorgio Natali, Paola Nisticò, Luisa Imberti

**Affiliations:** 1 Diagnostics Department, Spedali Civili di Brescia, 25123 Brescia, Italy; 2 Immunology Laboratory, Regina Elena Cancer Institute, via delle Messi d'Oro 156, 00158 Rome, Italy

## Abstract

**Background:**

Assessment of T-cell diversity, besides giving insights about the molecular basis of tumor antigen recognition, has clinical implications since it provides criteria for evaluating antigen-specific T cells clinically relevant for spontaneous and vaccine-induced anti-tumor activity. Melan-A is one of the melanoma antigens most frequently recognized by peripheral and tumor-infiltrating lymphocytes in HLA-A2+ melanoma patients. Many clinical trials involving anti-tumor vaccination have been conducted using modified versions of this peptide.

**Methods:**

We conducted an in-depth characterization of 210 T-cell receptor beta chain (TRB) clonotypes derived from T cells of HLA-A2+ melanoma patients displaying cytotoxic activity against natural and A27L-modified Melan-A peptides. One hundred and thirteen Melan-A-specific clonotypes from melanoma-free subjects, 199 clonotypes from T-cell clones from melanoma patients specific for melanoma antigens other than Melan-A, and 305 clonotypes derived from T cells of HLA-A2+ individuals showing unrelated specificities, were used as control. After sequence analysis, performed according to the IMGT definitions, TRBV and TRBJ usage, CDR3 length and amino acid composition were compared in the four groups of clonotypes.

**Results:**

TRB sequences of Melan-A-specific clonotypes obtained from melanoma patients were highly heterogeneous, but displayed a preferential usage of few TRBV and TRBJ segments. Furthermore, they included a recurrent "public" amino acid motif (Glycine-Leucine-Glycine at positions 110-112-113 of the CDR3) rearranged with dominant TRBV and TRBJ segments and, in one case, associated with a full conservation of the entire TRB sequence.

**Conclusion:**

Contrary to what observed for public anti-Melan-A T-cell receptor alpha motifs, which had been identified in several clonotypes of both melanoma patients and healthy controls, the unexpectedly high contribution of a public TRB motif in the recognition of a dominant melanoma epitope in melanoma patients may provide important information about the biology of anti-tumor T-cell responses and improve monitoring strategies of anti-tumor vaccines.

## Background

T-cell receptor (TR) plays a central role in the immune response, interacting with peptide antigens (Ags) and with major histocompatibility complex (MHC) molecules. TR alpha (TRA) and beta subunits are comprised of a variable (V) and a constant (C) amino acidic region. The TRBV region, referred according to the ImMunoGeneTics (IMGT) database [1], is encoded by V, diversity (D), and joining (J) gene segments. The juxtaposition of these segments [2], the lack of precision during V(D)J gene rearrangement and the removal and/or addition of non-template encoded nucleotides at V(D)J junctions [3], create a region of hypervariability known as complementarity-determining region 3 (CDR3).

Despite the potentially vast T-cell repertoire, restrictions of TR composition, known as TR bias, are commonly observed [4]. These TR constraints include the preferential usage of one TRV or TRJ region without conserved CDR3, the selection of conserved amino acids (up to five) or 'motifs' at the same CDR3 specific positions, and the selection of clonal TR sequences with identical CDR3 [4]. The different individual responses to discrete Ags are manifested in terms of personal, or "private", and shared, or "public", motifs in the TR sequences [4]. A private TR repertoire describes a situation in which T cells of distinct subjects responding to the same peptide-MHC complex have no significant overlaps in their TR sequences. In contrast, TR repertoires are defined public when Ag-specific T cells in several individuals use the same TR motifs, either in the TRA or TRB chains. To date, TRA and TRB public motifs have been described in human T-cell responses directed against viral peptides [4], while, in the anti-melanoma Ag response, only public TRA motifs have been reported [5-7]. However, TRA constraints, in particular within TRAV12-1 (previously defined Vα2 or TCRAV2.1) T cells, were observed not only in melanoma patients [5-7], but also in cord blood, thymocytes and PBL of non tumor-bearing controls [5], as well as in several subjects with *vitiligo *[8,9]. On the contrary, no public TRB motifs were identified in the sequences of Melan-A-specific T cells of melanoma patients and controls [5-8,10-19]. The unreported identification of public TRB in anti-melanoma Ag response may be related to the use of different methodological approaches employed to obtain T-cell lines or clones and to analyze CTL activity, as well as to prepare, characterize and analyze TR sequences. Another explanation can be the low number of patients analyzed in different studies. To bypass these limitations we took advantage, in the present study, of the availability of several published and unpublished TRB sequences obtained from a number of melanoma patients in order to study different aspects of TRB chain structural constraints imposed by the melanoma Ag MART1/Melan-A (hereafter reported as Melan-A). This differentiation Ag is a membrane-embedded protein of 118 amino acids expressed both by melanocytes and melanoma cells. Among the melanoma-associated Ags identified so far, Melan-A has received particular attention because of its immune dominance in HLA-A2+ patients. A large number of T-cell clones generated from HLA-A2+ patients are cross-reactive against either the natural nonamer/decamer Melan-A peptide (26/27–38) or the Alanine-to-Leucine substituted heteroclitic Melan-A A27L peptide [20,21]. Here, we identified several melanoma/HLA-A2-restricted TRB clonotypes (sequences showing different CDR3 in a given individual), and, after the definition of a common TR nomenclature, numbering and CDR3 designation, we studied in details their molecular features.

## Methods

The TRB sequences analyzed in this study were obtained either from previously reported or still unpublished studies. The rationale underlying selection of the 4 groups of TR sequences was to take into account three characteristics of the TR clonotypes which may generate biases in the selection of CDR3 region, *i.e*. Melan-A specificity, HLA-restriction and categories of individuals analyzed. Two hundred and ten Melan-A-specific clonotypes [[5-7,10-18] and manuscript in preparation], sequenced starting from T-cell lines or clones obtained from PBL and/or tumor-infiltrating lymphocytes (TIL) of melanoma patients ("Mel/M-A" group; Table 1), were compared with 113 Melan-A-specific clonotypes ("Ctrl/M-A" group) from healthy controls and from a subject with *vitiligo *[5,8,19], 199 clonotypes specific either for melanoma Ags other than Melan-A peptide or with undetermined specificity ("Mel/noM-A" group) obtained from T cells of melanoma patients [22-41], and 305 clonotypes prepared from HLA-A2+ melanoma-free patients ("Ctrl/HLA-A2+" group) selected because sequenced from T-cell lines and clones displaying CTL activity against unrelated Ags [42-54]. One hundred and seventy clonotypes of the Mel/M-A group and 85 from the Ctrl/M-A group were specific for the HLA-A2-restricted A27L-modified Melan-A peptide and their CTL activity was evaluated using a multimer-based approach [[5,6,8,12-14,17-19], and manuscript in preparation], by competition assay [15], or by analyzing the production of IL-2 in response to HLA-A2 Melan-A-expressing melanoma cell lines [7]. The remaining 40 clonotypes derived from cells of melanoma patients displayed CTL activity against natural Melan-A peptide, as demonstrated by ^51^Cr release assay [10,11,16]. Twenty-eight clonotypes of the Ctrl/M-A group, although specific for Melan-A peptide, were obtained from HLA-A2-negative healthy controls. Details on type of treatment, including vaccination, the starting material (peripheral blood or TIL), the experimental procedures used to obtain T-cell lines and clones or to analyze CTL activity, as well as the methodologies for TR sequencing are specified in the references included in Table 1. Before analysis, sequences available only in nucleotide form were translated into their amino acidic counterparts. All sequences analyzed in this study are supplied in the supplemental tables (additional file 1, 2, 3 and 4) showing, respectively, the clonotypes from Mel/M-A, Ctrl/M-A, Mel/noM-A and Ctrl/HLA-A2+ groups. In order to obtain uniformed information, TRBV gene family and CDR3 amino acid positions were named and numbered according to the IMGT indications [55].

**Table 1:** Characteristics of the TR clonotypes analyzed in this study

	*number of*										
									
	***Clono type***	***seq*^*a*^**	***subjects/patients (patients ID)*^*b*^**	***HLA***	***vaccination***		***source***	***type of sequenced cells***	***specificity*^*c*^**	***TRBV selection***	***references***
**Mel/M-A**	47	90	5 (8,22, 15, 30, 38)	A2	modified Melan-A	pre/post^d^	PBL	T-cell clones	Melan-A*	no	in preparation
	6	6	1 (VER)	A2	no		PBL	T-cell clones	Melan-A*	no	5
	26	27	3 (M199, M180, M138)	A2	no		TIL	T-cell clones	Melan-A *	no	6
	11	11	10 (Mela01, 02, 03, 04, 05, 06, 10, 13, 15, 16)	A2	modified Melan-A CTL clones	pre	PBL	T-cell clones	Melan-A***	no	7
	2	17	2 (-)^e^	A2	no		TIL	CTL lines	Melan-A****	4, 28	10
	30	119	3 (1, 2, 3)	A2	no		PBL/TIL	CTL lines	Melan-A****	7, 20, 29, 12, 5	11
	18	54	2 (LAU 181,203)	A2	no		TIL	CD8-sorted cells	Melan-A*	27, 30	12
	7	7	3 (NW28, 29, 30)	A2	Melan-A, Tyrosinase, gp100	pre/post	PBL	CD8-sorted cells	Melan-A*	no	13
	27	50	1 (-)	A2	no		PBL/TIL	T-cell clones	Melan-A*	no	14
	9	10	3 (SK9-AV, M77, LB373)	A2	no		PBL/TIL	T-cell clones	Melan-A**	no	15
	8	9	5 (8959, LB39, AV, 501, 9742)	A2	no		PBL/TIL	T-cell clones	Melan-A****	no	16
	12	27	1 (LAU444)	A2	modified Melan-A	pre/post	TIL/PBL	CD8-sorted cells	Melan-A*	6, 28	17
	7	17	1 (LAU337)	A2	Melan-A	post	PBL	T-cell clones	Melan-A*	no	18
	**210**	**444**									
**Ctrl/M-A**	53	53	3 (HD421, HD009, T12)	A2	NA		PBL/Thymocytes	T-cell clones	Melan-A*	no	5
	32	37	1 (PSA)	A2	NA		PBL	T-cell clones	Melan-A*	no	8
	28	28	4 (HD001, HD002, HD010, CB886)	Various A2-	NA		PBL	CD8-sorted cells	Melan-A*	no	19
	**113**	**118**									
	4	8	1 (-)	A2, A24	peptide-pulsed DC^f^	post	PBL/TIL	-	-	no	22
**Mel/noM-A**	1	-	Patient 1	A11, A32	IL-7^+ ^autologous melanoma cells	pre/post	PBL/TIL/DTH	-	-	27	23
	2	2	1 (FON)	A2, A29	no		TIL	T-cell clones	autologous melanoma	no	24
	4	9	1 (MZ2)	Cw16	MNNG-treated melanoma cells	pre/post	PBL	T-cell clones	BAGE, MAGE1	no	25
	7	140	1 (-)	-	no		TIL/PBL/Skin	-	-	14, 29, 23	26
	9	40	1 (-)	B14	no		TIL/Tissue	T-cell clones/lines/TIL	-	6	27
	25	42	6 (20113, 20297,20254, 20249, 20360, 20063)	-	DNP-modified melanoma cells	post	TIL in metastases	-	-	no	28
	6	38	1 (til 620)	-	no		TIL	T-cell colture	Melan-A/gp100	20, 19, 13	29
	52	87	4 (1, 2, 5, 6)	A2	no		TIL, PBL, normal skin	-	-	27, 9, 20, 29, 28, 7	30
	11	42	1 (2)		autologous stem cells after CTX	pre/post	PBL	-	-	2	31
	3	3	2 (BON, MAR)	A2, A25	no		TIL	-	-	28, 2, 24	32
	3	3	1 (MZ2)	A1	autologous melanoma cells		PBL	T-cell clones	MAGE1	no	33
	5	10	1 (9742)	A2	no		PBL/TIL	T-cell clones, PBL-PHA	autologous melanoma	no	34
	19	38	1 (JB)	A1, A28	DNP-modified melanoma cells	post	TIL	-	autologous melanoma	27	35
	4	15	2 (1200, 501)	A1, A2	no		TIL	bulk/CTL microcultures	A1/A2+ melanoma cells	no	36
	22	172	3 (1622, 1464, 1214)	A24, 26; A3, 11, A24	no		Tissue	-	-	6, 27, 28, 24, 10	37
	1	1	1 (0831)	A2	no		TIL	-	-	8	38
	16	100	5 (1, 2, 3, 4, 6)	-	no		Tissue	-	-	4, 28, 25, 29	39
	1	15	1 (LB256)	A2	no		PBL	T-cell clones	gp100	no	40
	4	192	1 (1803)	A1	no		TIL	bulk + cultures	-	20	41
	**199**	**957**									
**Ctrl/HLA-A2+**	41	46	15 (BD, CL, DD, DP, HL, JE, JM, JN, JW, KD, KE, MO, MP, NM, SW)	A2	NA		PBL	T-cell clones	M58-66 (flu)	19	42
	56^g^	606	12 (PB1, PB2, PB3, PB4, RA1, RA2, RA3, RA4, RA5, RA11, RA14, RA15)	A2	NA		PBL/SFL	T-cell clones/CD8-sorted lines	GLC/A2 (EBV)	2, 20, 29, 9, 14	43
	9	9	2 (FM, JM)	A2	NA		PBL	T-cell lines	M57-68 (flu)	no	44
	42	-	5 (B, F, M, P, T)	A2	NA		PBL	CTL/CD8-sorted population	GL9 (EBV)	no	45
	79	-	9 (D, F, H, K, M, N, P, R, S)	A2	NA		PBL	CTL/CD8-sorted cells	NV9 (CMV)		45
	33	43	4 (BMT, HD, RA, KT)	A2	NA		PBL/SFL	T-cell clones	pp65 (NLV/A2, HCMV)	no	46
	5	92	3 (003, 065, 868)	A2	NA		PBL	T-cell lines/clones/CD8-sorted cells	GAG (HIV), POL(HIV)	28, 5, 12	47
	1	7	1 (HEU)	A2	NA		TIL	T-cell clones	lung cancer antigen	no	48
	3	31	1 (HEU)	A2	NA		TIL/PBL	T-cell clones	alpha-actinin-4	no	49
	14	28	2 (-, 5H13)	A2	NA		PBL	T-cell clones	mHag HA-2	no	50
	9	15	1 (2)	A2	NA		PBL	CD8-sorted cells	19-kDa M. tuberculosis	no	51
	6	9	1 (-)	A2	NA		TIL	T-cell clones	various tumor epitopes	no	52
	2	29	1 (LB37)	A2	NA		PBL	CD8-sorted cells	mutated malic enzyme	no	53
	5	24	2 (MS2, MS7)	A2	NA		PBL	T-cell culture	TALpep	no	54
	**305**	**939**									

^a ^Number of sequences from which the clonotypes have been selected.

^b ^Abbreviations: CTL: Cytotoxic T Lymphocytes; CTX: Chemotherapy; DNP: Dinitrophenyl; DTH: delayed-type hypersensitivity site: ID: Identification number; MNNG: N-methyl-N'-nitro-N-nitrosoguanidine; NA: not applicable; Seq: sequences; SFL: synovial fluid lymphocytes; TIL: Tissue infiltrating lymphocytes.

^c ^CTL specificity against modified Melan-A analyzed by *multimers; ** competition assay; *** production of IL-2 in response to HLA-A2 Melan-A-expressing melanoma cell lines **** or CTL specificity against natural Melan-A analyzed by ^51^Cr release assay.

^d ^Clonotypes identified either in pre or in post vaccination.

^e ^-: data not available.

^f ^MAGE-4, MAGE-10, GnTV, gp100, Melan-A, FluMP, FluBNP-pulsed dendritic cells.

^g ^Identical clonotypes are included if found in different patients.

Bold: total number of clonotypes and of sequenced TRBV chains in each group

### Statistical analysis

To analyze TRBV or TRBJ segment usage, the 95% confidence intervals of the respective proportions were calculated. "Preferentially used" were defined those segments whose lower limit of the respective 95% confidence interval was higher than the mean percentage of TRBV or TRBJ transcripts usage, obtained by arbitrarily hypothesizing a uniform distribution of all segments. When proportions were compared, Fisher's exact test was employed, while the differences between the means of CDR3 length distributions in the four groups of clonotypes were evaluated by Kruskal-Wallis test and Dunn's post-hoc test. Results were considered significant for p < 0.05.

## Results

### Preferential TRBV and TRBJ usage in HLA-A2/Melan-A restricted response in melanoma patients

**Figure 1:**
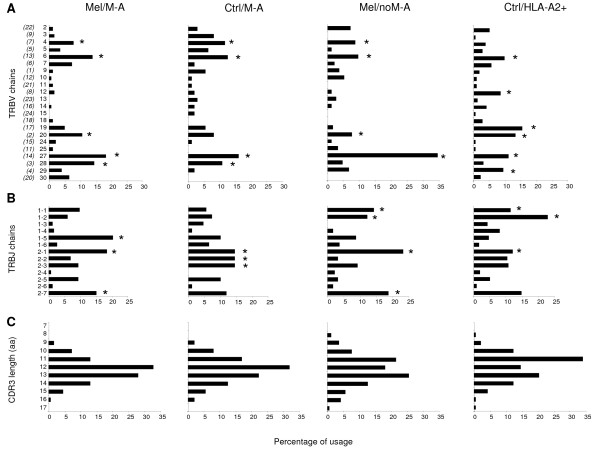
**TRB segments usage**. TRBV (**A**) and TRBJ (**B**) segments usage and CDR3 length (**C**) in clonotypes prepared from Melan-A-specific CTL lines and/or clones of melanoma patients (Mel/M-A), clonotypes from Melan-A-specific CTL of healthy controls and of a patient with vitiligo (Ctrl/M-A), clonotypes of melanoma patients specific for melanoma Ags other than Melan-A or with unknown specificity (Mel/noM-A), clonotypes from HLA-A2+ subjects derived from T lymphocytes specific for Ags unrelated to melanoma (Ctrl/HLA-A2+). The sequences analyzed here are those reported in Table 1. As indicated in Table 1, in some papers a pre-selection of cells bearing some specific TRBV segments was done before sequencing. * TRBV and TRBJ chains preferentially used within clonotype groups. The TRB nomenclature used throughout the paper is that of Lefranc *et al *[1]; the nomenclature reported in parenthesis is that of Arden *et al *[49]. (aa): amino acids.

We first investigated whether clonotypes identified in HLA-A2+ melanoma patients with CTL specificity against Melan-A (Mel/M-A group) had a preferential usage of particular TRBV chains and whether these preferential TRBV were also predominantly utilized in the control (Ctrl/M-A, Mel/noM-A and Ctrl/HLA-A2+ groups) clonotypes. As shown in Figure 1A, multiple transcripts covering the majority of the TRBV families were observed in the 4 groups of clonotypes, although some TRBV segments were preferentially used. In particular, while TRBV6 and TRBV27 were highly represented in all groups of clonotypes, TRBV4 was overrepresented in response to melanoma Ags but not to unrelated Ags, TRBV19 was preferentially used in clones of HLA-A2+ control individuals, and TRBV28 appeared to be preferentially selected only by Melan-A-specific CTL. TRBV usage comparison among the 4 groups suggested that the proportion of clonotypes using TRBV27 chains was higher in Mel/M-A, Ctrl/M-A and Mel/noM-A sequences compared to Ctrl/HLA-A2+ clonotypes (p = 0.03; p = 0.004; p < 0.001), while TRBV28 was significantly more frequent in Mel/M-A clonotypes than in Mel/noM-A and Ctrl/HLA-A2+ groups (p = 0.001 and p < 0.001).

Among Mel/M-A clonotypes there was a high number of clonotypes bearing the TRBJ2-1, TRBJ2-7 and TRBJ1-5 segments (Figure 1B). However, the first two TRBJ chains, however, were highly utilized also in other groups of clonotypes (Figure 1B), and had also been frequently observed among peripheral blood T-cells from healthy individuals [56].

The mean CDR3 length was highly similar (p = NS) in Mel/M-A, Ctrl/M-A and Mel/noM-A groups (mean ± SD: 12.37 ± 1.29, 12.32 ± 1.43 and 12.35 ± 1.71, respectively), but significantly lower in Ctrl/HLA-A2+ clonotypes (11.95 ± 1.50) in respect to Mel/M-A (p < 0.01) and Mel/noM-A sequences (p < 0.05). Furthermore, the majority of Mel/M-A and Ctrl/M-A CDR3 were 12 amino acid long (32.9% and 31.9% respectively), while most of CDR3 of Mel/noM-A and Ctrl/HLA-A2+ sequences were 13- and 11-amino acid-long, respectively (Figure 1C).

Collectively, the present analysis demonstrated that in melanoma patients there is a biased T-cell response to Melan-A, which is characterized by TR clonotypes using preferentially TRBV28 and TRBJ1-5 segments and containing a 12-amino acid-long CDR3.

### Public TRB CDR3 motif within HLA-A2/Melan-A-restricted clonotypes of melanoma patients

**Figure 2:**
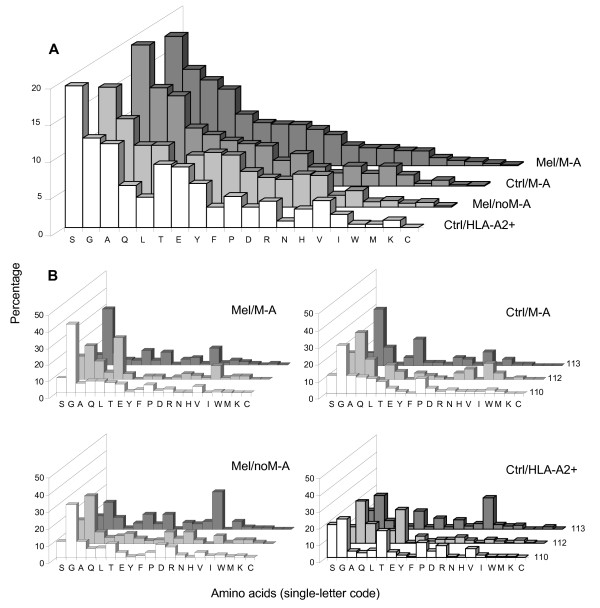
**Amino acid frequency**. Amino acid frequency in the entire IMGT-defined CDR3 (**A**) and in the position 110, 112 and 113 of the CDR3 (**B**) in the indicated groups of sequences.

**Figure 3:**
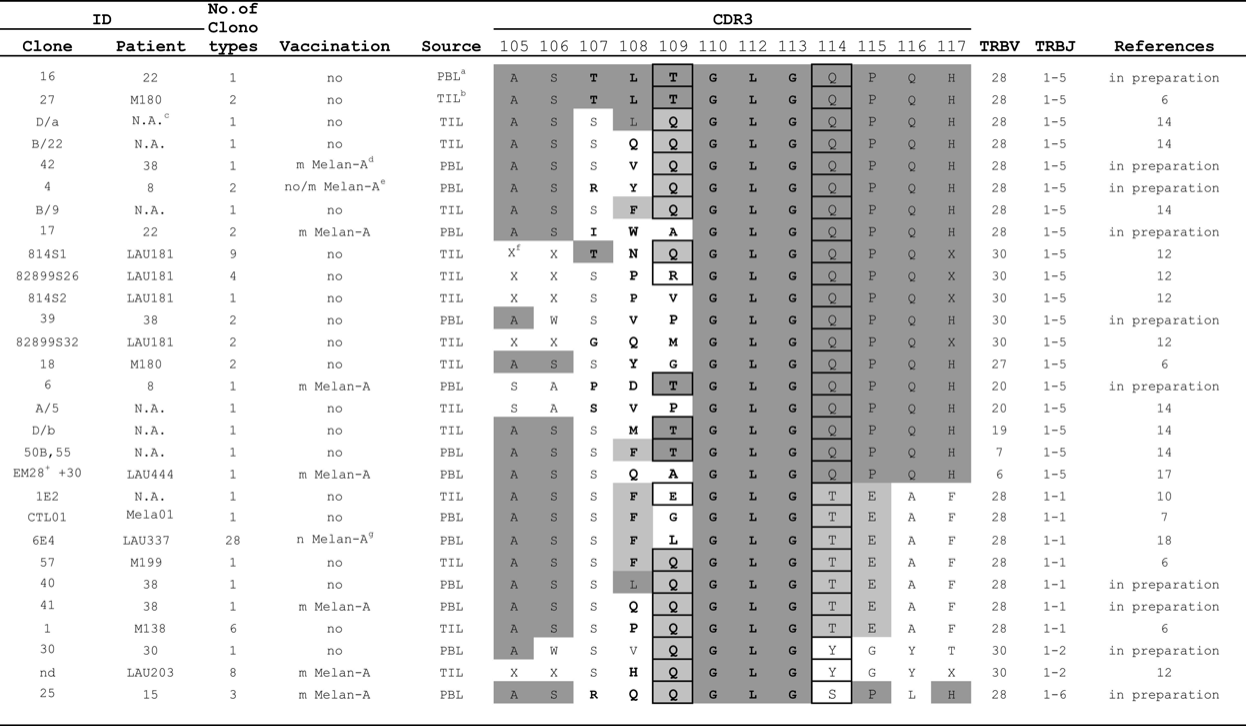
**Public motifs in Melan-A-specific clonotypes**. Aminoacidic composition and sequence alignments of public CDR3 of Melan-A-specific clonotypes found in melanoma patients. ^a^PBL: peripheral blood lymphocytes; ^b^TIL: tumor infiltrating lymphocytes; ^c^NA: ID not available; ^d^m: modified Melan-A A27L; ^e^Clonotype 4 was obtained from one T- clone was obtained before and one after vaccination; ^f^X: amino acid not available; ^g^n: natural Melan-A. In dark gray: amino acids identical to the consensus sequences; in light gray: other preferentially used amino acids at the given position; in bold: amino acids belonging to N-D-N region; in the boxes: hydrophilic amino acids at position 109 and 114.

The amino acid composition of TRB hypervariable regions of Melan-A-specific CTL from melanoma patients were subsequently analyzed in detail. Serine, Glycine, Alanine and Glutamine were by far the most frequently used residues in the IMGT-defined CDR3, and were almost equally represented in all groups of analyzed sequences (Figure 2A). However, while Alanine, Serine, and Glutamine were abundantly present because of their occurrence at positions 105, 106, 107 and 114 in the majority of canonical TRBV and TRBJ chains, Glycine, as reported for murine [57] and human sequences [56], was clearly predominant in the region created by N-D-N recombination events. Furthermore, in the N-D-N region of Mel/M-A and Ctrl/M-A sequences there was an increased Leucine usage (Figure 2A), and Glycine and Leucine were overrepresented at CDR3 positions 110, 112 and 113 (Figure 2B). Moreover, the overall percentage of non-polar amino acids at these CDR3 positions in the clonotypes carrying 12-amino acid-long CDR3s, which were the most commonly represented among the Melan-A-specific T-cell clones, was significantly higher in the Mel/M-A group (75%) compared to Ctrl/M-A (62%, p = 0.017), Mel/M-A (52%, p < 0.001) and Ctrl/HLA-A2+ (38%, p < 0.001) groups. This indicates that non-polar amino acids may be important for Melan-A-peptide-TR interaction. Furthermore, we found a public clonotype identified in two laboratories from cells of two melanoma patients: one was sequenced in our laboratory starting from a T-cell clone (ID 16) obtained from patient 22 [manuscript in preparation], the other from a T-cell clone (ID 27) obtained in the laboratory of Trautmann *et al *[6] employing melanoma-infiltrating lymphocytes of patient M180 (Figure 3). Both sequences contained identical 12-amino acid-long CDR3s, created by the joining of TRBV28 and TRBJ1-5 segments and containing a Glycine-Leucine-Glycine stretch at positions 110-112-113 of the CDR3. This motif was recurrent among other sequences derived from several patients, since it was found in 27 additional clonotypes sequenced in different laboratories and obtained from 15 melanoma patients. This peculiar motif rearranged only with members of TRBJ1 cluster, because 19 out of 29 clonotypes were joined with TRBJ1-5 segments, 7 with TRBJ1-1, 2 with TRBJ1-2 and one with TRBJ1-6 (Figure 3). TRBV usage was also restricted in these clonotypes since 16 of them were TRBV28, 7 were TRBV30 and 2 were TRBV20. The recurrent motif was found in Melan-A-specific CTL isolated from PBL and from tumor sites of HLA-A2+ melanoma patients, independently of the stage of disease and of the methodological approaches used for T-cell cloning. The same motif was identified in two Melan-A T-cell clones derived from cells of healthy donors [5,19], but not in the remaining 504 clonotypes sequenced from T-cell lines or clones with specificity for other Ags. Similarly, the Glycine-Leucine-Glycine motif at position 110-112-113 was absent in the 219 clonotypes identified analyzing 353 sequences randomly obtained from CD8+ lymphocytes of healthy subjects (data not shown). Furthermore, no common motifs were found when Melan-A-specific sequences of melanoma patients were compared using particular BV or BVBJ combinations. Of clinical relevance, the Glycine-Leucine-Glycine motif was detected in lymphocytes obtained from untreated patients, representing spontaneous anti-tumor responses, as well as from patients having undergone vaccination with the natural or modified peptides (Figure 3). Interestingly, one clonotype sequenced in our laboratory (ID 4) was detected both in samples prepared before and after the vaccination [58]. Furthermore, all but one clonotype containing the Glycine-Leucine-Glycine motif were sequenced from T-cell clones whose specificity was identified using modified Melan-A peptide/multimers. The specificity of the remaining clone for natural Melan-A peptide was established by the analysis of the ability of Melan-A-transfected COS-7 cells to stimulate IFN-γ release. This last clonotype (ID 1E2), identified by Cole *et al *[10], bore TRBV28 and TRBJ1-1 chains and differed only by the amino acid at position 109 (Figure 3) from ID 57, ID CTL01 and ID 6E4 clonotypes [6,7,18], which were sequenced starting from 3 melanoma patients. Furthermore, the same motif was present, at slightly different positions of the CDR3, in 7 other Melan-A-specific clonotypes [5,7,10,19], but never in non-Melan-A clonotypes. While the Glycine-Leucine-Glycine stretch is composed exclusively by non-polar or frankly hydrophobic amino acids, all the amino acids at position 114 and several of those at position 109 were hydrophilic (Figure 3). Finally, we looked for very similar sequences at the same CDR3 positions because it is conceivable that these sequences adopt equivalent structures in the recognition complex. We found a Glycine-Valine-Glycine stretch in 8 clonotypes, 5 of which were identified in melanoma patients [[4,12,14,30] and manuscript in preparation] and 3 in controls [3,5].

Since previous studies focusing on the analysis of shared TR amino acid sequences in humans did not address the extent to which TRB nucleotides are shared among public amino acid stretches, we identified the N-D-N regions of the 22 available nucleotide sequences of clonotypes with Glycine-Leucine-Glycine at position 110, 112 and 113. As summarized in Table 2, all N-D-N regions were different, with the only exception of those of ID D/a and ID 30 sequences, in which, however, the Adenine at the extreme 3'V region must be ascribed to the TRBV segment in clone ID D/a and to the D region in clone ID 30. Finally, the alignment of the 22 nucleotide sequences with the TRBV, TRBJ and TRBD germline gene segments allowed us to calculate the germline contribution and the number of nucleotide deletions (the so-called "nibbling") and additions during the VDJ recombination process. The exonucleolytic nibbling was highly heterogeneous: at 3' V end varied from 0 to 7 nucleotides, at 5' J end ranged from 4 to 9, at 3' D from 0 to 9 and at 5' D from 0 to 7. Similarly, N-addition was highly different at both sites ranging from 0 to 9 nucleotides at N1 and from 0 to 6 at N2 position. Finally, also TRBD region length is diverse since it varies from 3 to 8 nucleotides.

**Table 2:** Nucleotide composition of available N-D-N regions of public Melan-A-specific clonotypes of melanoma patients

**Clone ID**	***3' V region***	***N1***	***P***	***D region***	***N2***	***5' J region***	***TRBV***	***TRBJ***	***References***
D/a	GCCAGCAGTTTA..			....CAGGGG..	CTGGGG	.......CAGCCCCAGCAT	28	1–5	14
30	GCCTGGAGTGT			...ACAGGGG..	CTGGGG	.....TATGGCTACACC	30	1–2	in preparation
50B,55	GCCAGCAGCTT...	CACTGGGCT		......GGGG..		.......CAGCCCCAGCAT	7	1–5	14
17	GCCAGCA.......	TCTGGG		....CAGGG...	CTCGGG	.......CAGCCCCAGCAT	28	1–5	in preparation
814S1	XXXXXXA....^a^	CGAAT		....CAGGGG..	CTCGGG	.......CAGCCCCAGXXX	30	1–5	12
82899S32	XXXXXX.....	GGGCAAAT		.......GGGGC	TCGGG	.......CAGCCCCAGXXX	30	1–5	12
A/5	AGTGCTAG...	TGTGCC		.......GGGGC	TCGGG	.......CAGCCCCAGCAT	20	1–5	14
25	GCCAGCAG......	ACA		...ACAGGGG..	TTGGG	.........TTCACCCCTCCAC	28	1–6	in preparation
814S2	XXXXXXAG...	CCCGGT		.....AGGG...	TTGGG	......TCAGCCCCAGXXX	30	1–5	12
82899S26	XXXXXXAGT..	CC		....CAGGGGGC	TCGG	......TCAGCCCCAGXXX	30	1–5	12
42	GCCAGCAGT.....	GT		...ACAGGGG..	CTCGG	......TCAGCCCCAGCAT	28	1–5	in preparation
D/b	GCCAGTAGTAT...			..GACAGGG...	CTAGGG	.......CAGCCCCAGCAT	19	1–5	14
6	AGTGC.......	GCCCGAT		...ACAGGG...	CTTGGC	.......CAGCCCCAGCAT	20	1–5	in preparation
4	GCCAGCAG......	ATACCA		GGGAC.......	TAGGA	.......CAGCCCCAGCAT	28	1–5	in preparation
39	GCCTGGAGTGT	CC		....CAGGG...	CTAGG	......TCAGCCCCAGCAT	30	1–5	in preparation
NA	XXXXXXAGT..	CAT		....CAGGG...	ATTGGG	....CTATGGCTACXXX	30	1–2	12
16	GCCAGCA.......	CCCT		..GACAGGG...	CTTGGA	.......CAGCCCCAGCAT	28	1–5	in preparation
6E4	GCCAGCAGTTT...	TCT	C	GGG.........	TTGGG	....CACTGAAGCTTTC	28	1-1	18
40	GCCAGCAGTTTA..			....CAGGG...	TTGGGG	.....ACTGAAGCTTTC	28	1-1	in preparation
B/22	GCCAGCAGT.....	CA		...ACAGGG...	TTTGGG	......TCAGCCCCAGCAT	28	1–5	14
41	GCCAGCAG......	CCA		...ACAGGGG..	CTCGG	....CACTGAAGCTTTC	28	1-1	in preparation
B/9	GCCAGCAGTTT...	TCA		GGGAC.......	TCGG	......TCAGCCCCAGCAT	28	1–5	14

^a^Nucleotides not available

## Discussion

T-cells recognize peptide Ags in the context of MHC molecules through their TR, and during chronic infections, autoimmunity and alloreactivity a preferential use of particular TRA or TRB regions has been observed [4]. Therefore much effort has been put into the characterization also of tumor Ag-specific TRs. Several data demonstrated a major role of TRAV than TRBV chains in TR-Ag recognition, due to the higher number of contacts of this chain with peptides [59], and, accordingly, a preferential usage of a TRAV chain has been observed in Melan-A-specific T cells from melanoma or *vitiligo *patients and healthy donors [5-9]. However, this has not been considered a result of TR repertoire narrowing due to affinity focusing during Ag-driven immune responses, but to reflect a structural constraint already present in the pre-immune TR repertoire [5,9]. Differently from TRAV, the TRBV repertoire of Melan-A-specific T lymphocytes appears to be large and diverse in terms of clonal composition and TRBV region usage, as multiple clonotypic transcripts, covering the majority of the TRBV families, have been identified in HLA-A2+ patients [5-7,14,17]. Conversely, other authors reported that the recognition of melanoma Ags involved the use of T lymphocytes bearing specific TRBV chains, such TRBV5, TRBV9, TRBV19, TRBV27, and TRBV28 [16,18,23,30,35]. The different results are likely due to intrinsic limitations imposed by the limited number of patients analyzed and by the fact that the mature TR repertoire is influenced not only by the coding potential of TR VDJ regions, but also by the immunological history of the individuals. To clarify this issue, we analyzed several HLA-A2/Melan-A-specific clonotypes derived from 40 melanoma patients and we compared their features with those found in 103 other individuals including 8 subjects of Ctrl/M-A group, 36 of Mel/noM-A group and 59 of Ctrl/HLA-A2+ group. This comparative analysis indicated that T cells reacting with melanoma Ags utilize preferentially TRBV27 chain, but this segment is also predominant in clonotypes with unrelated specificity derived from HLA-A2+ individuals. On the contrary, TRBV28 chain is significantly more represented in HLA-A2+/Melan-A-specific T-cell clones obtained from melanoma patients and controls. It is of note that TRBV27 and TRBV28 chains (previously defined TCRBV14S1 and TCRBV13S1, respectively) were expressed at very low percentage when PBL of healthy individuals were analyzed by cytofluorimetry using a panel of TRBV subfamily-specific mAbs covering about 65% of TR-expressing cells [60]. Although we cannot exclude that anti-TRBV27 and anti-TRBV28 mAbs may not recognize well these TRBV chains, the overexpression of these segments in the clonotypes that we have analyzed strongly suggests that these TRBV segments are important for melanoma Ag recognition, with TRBV28 being preferentially involved in the interaction between TR and Melan-A.

Looking in depth at the peculiar features of TR-Melan-A interaction, we found a biased utilization of TRBJ1-5 segment and a 3-amino acid-long Glycine-Leucine-Glycine public motif occurring in several clonotypes of melanoma patients. Further biases were the frequent association of this public motif with TRBV28 and TRBJ1-5 segments and the lack of rearrangement with members of TRBJ2 cluster. The finding of this public motif demonstrates that the discrepancy between the anti-viral and anti-melanoma Ag responses is only apparent and supports our hypothesis that the lack of common TRB constraints among patients analyzed in different studies [5-8,10-18] is likely due to the paucity of individuals studied and to the diverse technical approaches employed for the sequence analysis. Indeed, Mandruzzato *et al *[14] have previously identified the Glycine-Leucine-Glycine stretch, but they could not appreciate the frequency of this feature since they studied a single melanoma patient. Clones carrying recurrent motifs were present at low frequency in each patient, exception made for two patients from whom 9 and 28 clones with the same Glycine-Leucine-Glycine-containing TR were isolated [12,18], while during viral infections, public clonotypes are very frequent not only within the population, but are also sequenced in a large number in the same patient [4,43-46]. This is not surprising since most of these studies were carried out in the context of chronic, most likely lifelong, viral infections, *i.e*. EBV infection, where exposure to Ags is continuous and a selective pressure on T cells remains constantly high.

There is not a general rule that could account for the occurrence of public T-cell responses. Some public TRB motifs have been made from near-germline recombination events, involving only few nucleotides deletion from V, D and J germline and no or minimal random nucleotide additions [61,62] but the extent of exonucleolytic nibbling and the substantial number of nucleotide additions in the public anti-Melan-A TR stretch exclude that its public nature is generated by near-germline rearrangements.

Looking at the biochemical structure of the public motif identified, one may speculate that the Glycine-Leucine-Glycine stretch positioned in the central region of the CDR3, which is surrounded by hydrophilic residues, can favour the interaction with the antigenic Melan-A peptide, which has a similar central Glycine-Isoleucine-Glycine motif, with the large non polar side chain of the Isoleucine protruding extensively from the molecular surface [63]. The relevance of this and of other structural affinities in the two sequences, such as the potential interactions between the hydrophilic residues flanking their central positions, might be assessed with more confidence when further data on the recently crystallized TR-Melan-A-MHC complex [64] will be available, and the spatial relationships between Melan-A and CDR3 amino acids will became clearer.

## Conclusion

The finding of a conserved amino acid motif in the CDR3, together with the selective use of certain TRBJ and TRBV segments, indicates an important role of the TRB chain in fine-tuning TR affinity of Melan-A-specific T cells of melanoma patients and argues against the hypothesis that high affinity TRs against self-Ags, like Melan-A, are removed during selection in the thymus or, alternatively, by tumor-induced deletion of dominant TR clonotypes [65].

Further studies are needed to elucidate the clinical relevance of these melanoma-associated clones, which were found not only in T-cell clones isolated from PBL but also from tumor sites, thus suggesting some lymph-node homing properties of the T cells bearing the public motif. However, whatever the function of these clonotypes is, the occurrence of this public CDR3 sequence may have implications for the tracking of tumor Ag-specific T cells in different clinical settings. In particular, sensitive molecular approaches targeting TRBV28+TRBJ1-5+ cells bearing Glycine-Leucine-Glycine motif could be designed to immune-monitor Melan-A-specific responses in melanoma patients and to investigate whether the presence of this specific motif can provide prognostic information, contributing to the design of efficient anti-melanoma vaccines.

## Competing interests

The authors declare that they have no competing interests.

## Authors' contributions

FS, and AS have made substantial contribution in the acquisition and alignment of sequences, in the analysis and interpretation of data, and helped to draft the manuscript. LC, BP, PGN, and PN, have been involved in drafting and critically revising the manuscript. LI conceived and coordinated the study and draft the manuscript. All authors read and approved the final version of the manuscript.

## Supplementary Material

Additional file 1 **Supplemental table 1.** Table of TRB sequences of 210 clonotypes from Melan-A-specific T-cell lines or clones obtained from HLA-A2+ melanoma patients. Click here for file

Additional file 2 **Supplemental table 2.** Table of TRB sequences of 113 clonotypes from Melan-A-specific T-cell clones of subjects without melanoma Click here for file

Additional file 3 **Supplemental table 3.** Table of TRB sequences of 199 clonotypes from T-cell lines or clones obtained from melanoma patients with variable Ag-specificity and no known Melan-A restriction. Click here for file

Additional file 4 **Supplemental table 4.** Table of TRB sequences of 305 clonotypes from HLA-A2+ T-cell lines or clones with specificities unrelated to melanoma. Click here for file
